# Behaviours of general practitioners in the city of douala’s in cameroon in front of depressive disorders

**DOI:** 10.1192/j.eurpsy.2021.869

**Published:** 2021-08-13

**Authors:** T. Michael

**Affiliations:** Psychiatry, university of nairobi, nairobi, Kenya

**Keywords:** Practice, Obstacle, Depression, Diagnostic

## Abstract

**Introduction:**

In Cameroon, skepticism and neglect of doctors towards patients with mental disorders is noted. In order to change this, it was important to have an objective assessment. Depressive disorders are the most common form of mental disorders and Douala has the second largest number of general practitioners in the country. Thus, we proposed to evaluate the behaviour of general practitioners in Douala in front of patients with depressive disorders.

**Objectives:**

Evaluate the frequency of depressive disorders in outpatient general consultation Evaluate the competence of general practitioners at diagnosing depressive disorders Evaluate the practices of general practitioners towards depressive disorders Evaluate obstacles faced by general practitioners regarding depressive disorders

**Methods:**

We conducted a cross-sectional descriptive study from February to June 2017 in the outpatient department. For each general practitioner include, we had 3 patients who complete the patient health questionnaire to find out if the patient has depression. During each consultation, we filled out a clinical fact sheet to determine if the general practitioner had diagnosed a depressive disorder. If so, what care has he taken? Finally, we gave the general practitioner a questionnaire to know his difficulties when facing depression.

**Results:**

We obtained a frequency of 32.5% of depressive disorders in consultation of general medicine in Douala and a rate of diagnosis by general practitioners of 1.92%. Diagnosed cases have just received counseling.

**Conclusions:**

in Cameroon, despite the low interest in depressive disorders, they constitute a public health issue in Douala, and surely in Cameroon; Because of its frequency and the harm, they inflict on patients.

**Conflict of interest:**

I don’t have any conflict of interest

